# Supernumerary eumorphic mandibular incisor in association with aggressive periodontitis

**DOI:** 10.4103/0972-124X.70836

**Published:** 2010

**Authors:** Vikas Verma, Amit Goel, Mohd. Sabir

**Affiliations:** *Department of Periodontics, Teerthanker Mahaveer Dental College and Research Center, Moradabad, Uttar Pradesh, India*; 1 *Department of Periodontics, Aligarh Muslim University, Aligarh, Uttar Pradesh, India*

**Keywords:** Eumorphic, localized aggressive periodontitis, mandibular incisor, supernumerary

## Abstract

According to the literature, the prevalence of supernumerary teeth is 1% to 4% of permanent dentitions; and among these, the presence of fifth mandibular incisor — a supernumerary eumorphic tooth — has rarely been described in literature, and its association with localized aggressive periodontitis is an even more rare entity. This paper reports a very rare case of unusual association of supernumerary eumorphic fifth mandibular incisor with aggressive periodontitis in a Muslim individual, so that these findings generate curiosity and inspire others to carry out further studies and investigations.

## INTRODUCTION

The condition of supernumerary teeth, or hyperdontia [Online Mendelian Inheritance in Man-187100], is defined as an excess number of teeth compared to the normal dental formula, or existing of teeth additional to the normal series in the dental arches. Their classification is dependent on their position and form. Hyperdontia may occur as a single tooth or multiple teeth, unilateral or bilateral, or in one or both jaws. This classification morphologically can be subcategorized into eumorphic (supplemental) and dysmorphic (rudimentary) elements. Supernumerary eumorphic teeth have the same morphology as that of the normal teeth, whereas dysmorphic ones are small and conical, tuberculate or odontome in shape.[1–3]

Although there is no consensus on the etiology of supernumerary teeth, one etiologic theory suggests that the supernumerary tooth is created as a result of a dichotomy of the tooth bud.[4] Another theory, well supported in the literature is the hyperactivity theory, which suggests that supernumeraries are formed as a result of local, independent, conditioned hyperactivity of the dental lamina.[4,5] Heredity may also play a role in the occurrence of this anomaly. However, the anomaly does not follow a simple Mendelian pattern.[1,3,6–8]

In a survey of 2,000 school-going children, Brook found that supernumerary teeth were present in 2.1% of permanent dentitions.[9] The prevalence of supernumerary teeth was 2.97% as reported by Acikgoz *et al*.[10] The prevalence varies from 1% to 4%.[11] Prevalence of supernumerary teeth in mandibular incisor region is 2% of the total supernumerary prevalence,[12,13] and it is the lowest in the oral cavity.[12,14,15] Very few cases of 5 mandibular incisors have been reported in the literature.[3,16,17] The prevalence of aggressive periodontitis is about 0.1% as reported by Odell and Hughes;[18] 0.32%, by Lopez *et al*.;[19] and 0.76%, by Melvin *et al*.[20]

The possible association between supernumerary teeth and aggressive periodontitis has been reported in literature.[10,18,21] But there is no published report on eumorphic fifth well-aligned mandibular incisor associated with aggressive periodontitis. The purpose of this case report was to present a case of 5 mandibular incisors that are same in morphology and without fusion between crowns and roots of lower incisors and are associated with aggressive periodontitis.

## CASE REPORT

**Figure 1:**
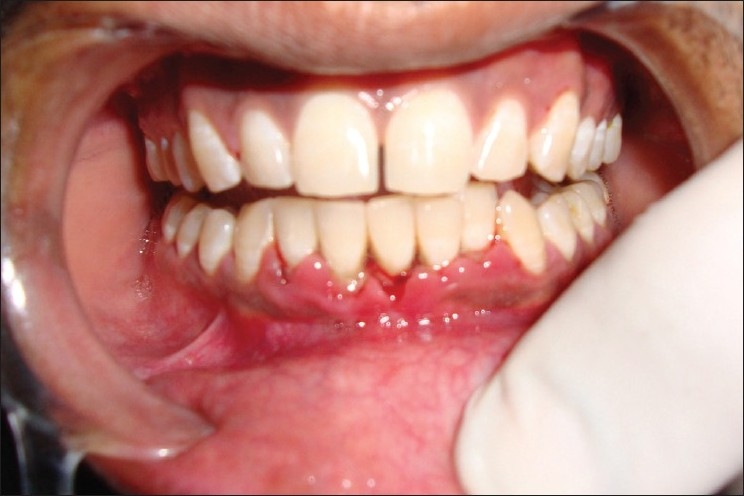
Clinical view showing morphologically similar 5 mandibular incisors

**Figure 2:**
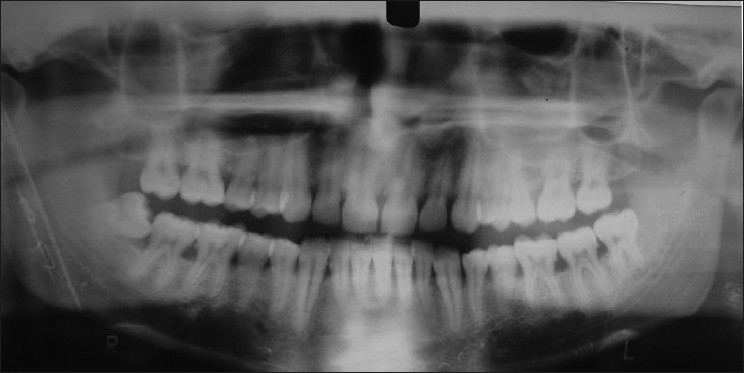
Orthopantomograph of subject showing 5 mandibularincisors and bone loss

**Figure 3:**
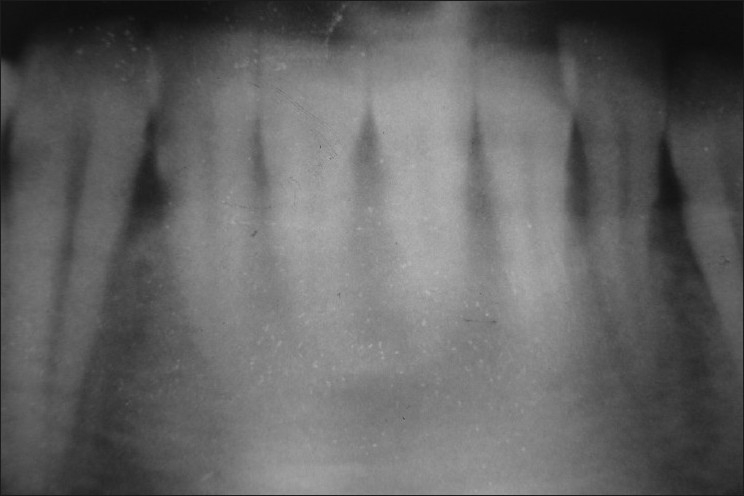
Closer view of orthopantomograph of the subject showing5 mandibular incisors

A 25-year-old Indian Muslim man reported to the Department of Periodontics, Teerthanker Mahaveer Dental College and Research Centre, Moradabad, with a chief complaint of bleeding gums and foul smell since 2 to 3 years. He was found to have localized aggressive periodontitis. On routine clinical and radiographic examination, supernumerary eumorphic fifth mandibular incisor was found. We could not differentiate the eumorphic fifth mandibular incisor from the remaining mandibular incisors, clinically or radiographically, neither was there any fusion between crowns and roots in lower incisors. The five mandibular incisors were separate and same in morphology [Figures 1–3]. Apart from this, no other case of supernumerary teeth was detected either in the subject or in his family members.

Localized aggressive periodontitis was diagnosed by ≥5 mm pocket probing depth around all the four first molars with moderate vertical bone loss radiographically. In lower incisors, there was ≥5 mm attachment loss but due to recession, and pocket depth was ≥3 mm.

In lower jaw, third molar of right side was impacted, and impaction was mesioangular, whereas the third molar of left side was well erupted and in normal occlusion. Hematological examination consisting of total leukocyte count (TLC), differential leukocyte count (DLC), hemoglobin (Hb), erythrocyte sedimentation rate (ESR), clotting time (CT), bleeding time (BT) revealed no significant findings.

## DISCUSSION

Prevalence of supernumerary teeth in mandibular incisor region is 2% of total supernumerary prevalence,[12,13] and it is the lowest in the oral cavity.[12,14,15] Previously prevalence rates of supernumerary eumorphic mandibular incisors have not been reported as there are very few reported cases of this tooth anomaly.[3,16] So, it is a very rare entity.

In the present case, supernumerary eumorphic mandibular incisor was normal, well individualized with no fusion in roots and crowns. Differentiation of this supplementary tooth from other mandibular incisors was difficult. This fifth incisor mimicked other mandibular incisors in morphology, radiographically and clinically. Hence this type of supernumerary tooth is overlooked most of the time, unless diagnosed by chance by a dentist during clinical and radiographic examination.

Supernumerary teeth in mandibular incisor region may be seen in some hereditary syndromes (gardener syndrome and acrofacial dystosis).[12,14] They were ruled out in our subject because of absence of any typical clinical features associated with these entities. Supernumerary eumorphic mandibular incisors may have a familial association. Cassia *et al*. reported the presence of a supernumerary eumorphic fifth mandibular incisor in a Lebanese consanguineous family where 4 individuals displayed 5 mandibular incisors with the same shape and size, and they hypothesized the possibility of an autosomal recessive inheritance for this nonsyndromic trait.[3] Sami *et al*. (2008) reported the case of homozygosity-mapping to identify a homozygous region with different alleles at chromosome 16q 12.2, located at the marker D16S415, which likely harbors the gene underlying this anomaly.[7] However, in the present case, no familial association was detected, i.e., no other family member had this anomaly or any other type of supernumerary tooth.

A possible association between supernumerary teeth and localized aggressive periodontitis has been described in a small number of reported cases.[10,18,21,22] Localized aggressive periodontitis is characterized by severe attachment and angular bone loss, particularly in incisors and molars. Aggressive periodontitis is reported in various study populations — 0.1%, by Odell and Hughes[18]; 0.32%, by Lopez *et al*.[19]; and 0.76%, by Melvin *et al*.[20] So, both the entities, viz., aggressive periodontitis and supernumerary eumorphic mandibular incisor, are uncommon conditions.

The first study recognizing the possible connection between supernumerary teeth and periodontitis was a case report by Eley in 1974.[21] In 1981, Rubin *et al*. described two identical Black twins with localized juvenile periodontitis, multiple supernumerary teeth and no dental caries. The authors hypothesized that all these three entities were due to genetic influence.[6] As Odell and Hughes reported in 1995, both aggressive periodontitis and supernumerary teeth are uncommon but have a familial tendency, and an association may be seen in a small minority of cases. Both entities show familial prevalence, but at the same time, both are consistent with multifactorial and multigenic etiology.[18] However, regarding correlation between aggressive periodontitis and supernumerary eumorphic mandibular incisor, it may be thought that some responsible genes are linked and expressed together. But in a retrospective study done by Gokhan *et al*. (2004), the association between aggressive periodontitis and supernumerary teeth was suggested to be a random rather than a biologic one.[10]

To conclude, one may think in terms of the correlation between aggressive periodontitis and supernumerary eumorphic mandibular incisor as in this study. This does not mean that both entities have biological connection. However, association between these two entities is definitely a rare one. To prove such biological connection, further studies and genetic investigations are required to be carried out.
